# Uncommon mutational profiles of metastatic colorectal cancer detected during routine genotyping using next generation sequencing

**DOI:** 10.1038/s41598-019-43646-0

**Published:** 2019-05-08

**Authors:** Claire Franczak, Shaun M. Kandathil, Pauline Gilson, Marie Husson, Marie Rouyer, Jessica Demange, Agnès Leroux, Jean-Louis Merlin, Alexandre Harlé

**Affiliations:** 1 Institut de Cancérologie de Lorraine, Service de Biopathologie, 54519 Vandoeuvre les Nancy, France; 2 Department of Computer Science, University College London, Gower Street, London, WC1E 6BT United Kingdom; 3 The Francis Crick Institute, 1 Midland Road, London, NW1 1AT United Kingdom; 4 Université de Lorraine, CNRS UMR 7039 CRAN, Institut de Cancérologie de Lorraine, Service de Biopathologie, 6 avenue de Bourgogne CS 30519, 54519 Vandoeuvre-lès-Nancy Cedex, France

**Keywords:** Colorectal cancer, Predictive markers

## Abstract

*RAS* genotyping is mandatory to predict anti-EGFR monoclonal antibodies (mAbs) therapy resistance and *BRAF* genotyping is a relevant prognosis marker in patients with metastatic colorectal cancer. Although the role of hotspot mutations is well defined, the impact of uncommon mutations is still unknown. In this study, we aimed to discuss the potential utility of detecting uncommon *RAS* and *BRAF* mutation profiles with next-generation sequencing. A total of 779 FFPE samples from patients with metastatic colorectal cancer with valid NGS results were screened and 22 uncommon mutational profiles of *KRAS*, *NRAS* and *BRAF* genes were selected. *In silico* prediction of mutation impact was then assessed by 2 predictive scores and a structural protein modelling. Three samples carry a single *KRAS* non-hotspot mutation, one a single *NRAS* non-hotspot mutation, four a single *BRAF* non-hotspot mutation and fourteen carry several mutations. This *in silico* study shows that some non-hotspot *RAS* mutations seem to behave like hotspot mutations and warrant further examination to assess whether they should confer a resistance to anti-EGFR mAbs therapy for patients bearing these non-hotspot *RAS* mutations. For *BRAF* gene, non-V600E mutations may characterise a novel subtype of mCRC with better prognosis, potentially implying a modification of therapeutic strategy.

## Introduction

Combination of targeted therapies like anti-EGFR monoclonal antibodies (anti-EGFR mAbs) with chemotherapy regimen (FOLFOX or FOLFIRI) improves progression-free (PFS) and overall survival (OS) in patients with metastatic colorectal cancer (mCRC)^1^. Tumor mutation hotspots associated with clinical resistance to anti-EGFR mAbs like *KRAS* exon 2 (codons 12 and 13), exon 3 (codons 59 and 61), exon 4 (codons 117 and 146) and *NRAS* exon 2 (codons 12 and 13), exon 3 (codons 59 and 61) and exon 4 (codons 117 and 146) are now well identified and are systematically assessed prior to anti-EGFR mAbs prescription^1–5^. KRAS and NRAS are both Ras serine-threonine kinases, located downstream of EGFR in the Ras/Raf/MAPK pathway. Mutations in these codons cause constitutive activation of the RAS-MAPK pathway. *KRAS* and *NRAS* mutations are reported in 40–50% and 5–8% of patients with mCRC, respectively^6^.

Tumor *KRAS* and *NRAS* mutational statuses are usually assessed using polymerase chain reaction (PCR)-based assays designed for detection of major hotspot mutations. Most PCR-based assays, by their design, are not able to detect non-hotspot mutations; thus, patients with a tumor bearing a non-hotspot mutation are labeled as “wild-type” even though a tumor mutation exists. Next generation sequencing (NGS) assays allow the analysis of full exons and are able to detect uncommon mutational profiles. The impact of non-hotspot mutations of *KRAS*, *NRAS* and *BRAF* on anti-EGFR mAbs resistance is still unclear and it may be useful to study their impact in patients with mCRC.

BRAF is a serine-threonine kinase, located downstream of EGFR in the Ras/Raf/MAPK pathway^7^. *BRAF* mutations are reported in 5–10% of patients with mCRC^3,6,8^. p.(Val600Glu) (V600E) is the main *BRAF* hotspot mutation^9^. This mutation, located on exon 15 of the *BRAF* protein kinase activation domain leads to an increase of BRAF activity, 130 to 700 times higher than in wild-type (WT) *BRAF*^10,11^. In mCRC, this hotspot mutation is recognized as a poor prognosis factor^9^. Non-hotspot mutations have also an impact on *BRAF* protein activity, leading to high, intermediate or impaired kinase activity^12,13^.

The aim of this study was to identify isolated or concomitant non-hotspot *RAS* and *BRAF* mutations detected during routine sequencing by NGS and discuss their potential role in treatment resistance and prognosis for patients with mCRC using *in silico* prediction tools.

## Results

We retrospectively collected data from 857 mCRC samples including 779 samples with valid NGS results. DNA quality was suitable for 91% of the samples for NGS and uncommon mutational profiles were reported in 22 (2.7% of total) samples.

The histological subtypes were: adenocarcinoma for 11 samples, liberkunhian adenocarcinoma for 9 samples, mucinous carcinoma for 1 sample, and ductal carcinoma for 1 sample. Twenty samples were from primary tumors and 2 were from liver metastases (Table 1).

**Table 1:** Uncommon mutational profiles found in our study and tumor features.

#	Primary tumor localization	Tumor lesion analyzed	Histological type	Gene	Exon	Nucleotidic variation	Protein variatio	COSMIC ID.^a^	Significance	Coverage	MAF (%)^d^
**#1**	Left colon junction	Primary	Lieberkuhnian adenocarcinoma	KRAS	2	c.34G > A	p.(Gly12Ser)	COSM517	Missense	2623	32.0
				NRAS	3	c.181C > A	p.(Gln61Lys)	COSM580	Missense	4133	14.4
**#2**	Sigmoid Colon	Primary	Mucinous carcinoma	KRAS	2	c.34G > A	p.(Gly12Ser)	COSM517	Missense	3412	25.4
				NRAS	2	c.38G > T	p.(Gly13Val)	COSM574	Missense	3009	12.3
**#3**	Right colon	Primary	Ductal carcinoma	KRAS	2	c.37G > T	p.(Gly13Cys)	COSM527	Missense	2694	57.9
**#4**	Sigmoid colon	Primary	Lieberkuhnian adenocarcinoma	KRAS	2	c.37G > T	p.(Gly13Cys)	COSM527	Missense	800	30.6
**#5**	Colon	Metastasis (liver)	Lieberkuhnian adenocarcinoma	KRAS	2	c.24A > G	p.(Val8Val)	COSM1360891	Silent mutation	1117	70.6
**#6**	Sigmoid colon	Primary	Adenocarcinoma	NRAS	4	c.360G > A	p.(Leu120Leu)	Not described*	Silent mutation	907	40.6
**#7**	Right colon	Primary	Lieberkuhnian adenocarcinoma	KRAS	4	c.360G > A	p.(Leu120Leu)	Not described*	Silent mutation	2688	48.0
				KRAS	4	c.353G > A	p.(Cys118Tyr)	Not described*	Missense	2688	14.4
				KRAS	4	c.418C > T	p.(Pro140Ser)	COSM4169136	Missense	2688	6.0
**#8**	Right colon	Primary	Adenocarcinoma	KRAS	4	c.344G > A	p.(Gly115Glu)	Not described*	Missense	1634	4.3
				NRAS	2	c.69A > G	p.(Leu23Leu)	*rs771113899* ^*#*^	Silent mutation	1819	1.8
**#9**	Sigmoid colon	Primary	Lieberkuhnian adenocarcinoma	KRAS	3	c.281G > A	p.(Arg68Arg)	Not described*	Silent mutation	1849	25.0
				KRAS	4	c.394G > A	p.(Asp132Asn)	Not described*	Missense	1836	4.0
				NRAS	2	c.64C > T	p.(Gln22)*	Not described*	Stop mutation	2913	8.0
#10	NA	Metastasis (liver)	Adenocarcinoma	NRAS	2	c.99T > G	p.(Asp33Glu)	Not described^b^	Missense	8161	22.5
				KRAS	2	c.35G > T	p.(Gly12Val)	COSM520^c^	Missense	2397	17.6
#11	Rectum	Primary	Adenocarcinoma	BRAF	15	c.1742A > G	p.(Asn581Ser)	COSM462	Missense	4048	27.6
				NRAS	2	c.34G > T	p.(Gly12Cys)	COSM562^c^	Missense	2398	15.7
#12	Right colon	Primary	Lieberkuhnian adenocarcinoma	KRAS	2	c.40G > A	p.(Val14Ile)	COSM12722	Missense	2887	14.5
				BRAF	15	c.1805C > T	p.(Ser602Phe)	Not described^b^	Missense	5208	22.6
				HRAS	3	c.217C > T	p.(Arg73Cys)	Not described^b^	Missense	2825	19
				MET	14	c.3050A > C	p.(Glu1017Ala)	Not described^b^	Missense	11344	14.3
#13	Colon	Primary	Lieberkuhnian adenocarcinoma	BRAF	11	c.1396G > A	p.(Gly466Arg)	COSM253328	Missense	5991	21.2
#14	Colon	Primary	Adenocarcinoma	BRAF	15	c.1781A > G	p.(Asp594Gly)	COSM467	Missense	11967	31.5
#15	Rectum	Primary	Lieberkuhnian adenocarcinoma	BRAF	11	c.1406G > T	p.(Gly469Val)	COSM469	Missense	6910	46.2
#16	Duodenum	Primary	Adenocarcinoma	BRAF	15	c.1780G > A	p.(Asp594Asn)	COSM27639	Missense	8067	21
				KRAS	2	c.38G > A	p.(Gly13Asp)	COSM532	Missense	3651	19.9
#17	Caecum	Primary	Lieberkuhnian adenocarcinoma	BRAF	11	c.1406G > C	p.(Gly469Ala)	COSM460	Missense	5570	31.4
				KRAS	2	c.35G > A	p.(Gly12Asp)	COSM521	Missense	736	50.8
#18	Rectum	Primary	Adenocarcinoma	BRAF	11	c.1397G > A	p.(Gly466Glu)	COSM453	Missense	12029	7.9
				KRAS	2	c.57G > T	p.(Leu19Phe)	COSM20818	Missense	12363	6.5
#19	Colon	Primary	Adenocarcinoma	KRAS	2	c.38G > A	p.(Gly13Asp)	COSM532	Missense	2083	25.6
				KRAS	4	c.436G > A	p.(Ala146Thr)	COSM19404	Missense	5445	25.6
				KRAS	3	c.264A > C	p.(Lys88Asn)	Not described^b^	Missense	6549	28.2
#20	Rectum	Primary	Adenocarcinoma	BRAF	11	c.1406G > C	p.(Gly469Ala)	COSM460	Missense	20393	31.3
				KRAS	4	c.351A > T	p.(Lys117Asn)	COSM28519	Missense	27295	16.8
#21	Rectosigmoid	Primary	Adenocarcinoma	BRAF	15	c.1801A > G	p.(Lys601Glu)	COSM478	Missense	9254	23.6
#22	Caecum	Primary	Adenocarcinoma	BRAF	15	c.1799T > A	p.(Val600Glu)	COSM476	Missense	12127	32.5
				KRAS	2	c.35G > T	p.(Gly12Val)	COSM520	Missense	9389	49.6

^a^As described in the Catalogue of Somatic Mutations in Cancer (COSMIC), available online at.

^b^Not described as somatic nor as single nucleotide polymorphism (SNP) in databases.

^c^*KRAS* and *NRAS* hotspot mutation.

^d^Mutant allele fraction (MAF).

The range of coverage for rare *KRAS*, *NRAS* or *BRAF* mutations was 700 to 27 000x. Mutant allele fraction was between 1.8% and 40.6% for non-hotspot *NRAS* mutations, between 4.0% and 14.5% for non-hotspot *KRAS* mutations and between 7.9% and 46.2% for non-hotspot *BRAF* mutations. Thirty-five mutations were missense mutations, 5 were silent mutations and one was a stop mutation.

All observed mutations are described in Table 1. Among 22 samples, 3 carried an isolated non-hotspot *KRAS* mutation (#3, #4, #5), 1 a *NRAS* mutation (#6) and 4 a *BRAF* non-hotspot mutation (#13, #14, #15, #21). Seven concomitant *RAS* mutations (#1, #2, #7, #8, #9, #10, #19) and 7 concomitant *RAS* and *BRAF* mutations (#11, #12, #16, #17, #18, #20, #22) were detected. One sample (#12) bore an *HRAS* mutation not previously described and no sample carried *MAP2K1* mutations. Twelve of these mutations are not yet described as somatic nor as single nucleotide polymorphism (SNP) in public database.

For each mutation, the PolyPhen-2 score, SIFT score, their interpretation, and protein domain impacted are described in Table 2. All *KRAS* and *NRAS* mutations observed in this study are located in the catalytic domain. The majority of these mutations are localized in the GTP binding site. For the *BRAF* gene, all mutations described in our study are found in the protein kinase domain. Predictions of the impact of each mutation are given in Table 2.

**Table 2:** SIFT, PolyPhen-2 score and protein localization of each identified mutation.

#	Gene	Exon	Nucleotidic variation	Protein variation	COSMIC ID.^a^	Significance	SIFT Score	SIFT score interpretation Predicted to be	PolyPhen-2 score	PolyPhen-2 score interpretation Predicted to be	FoldX ∆∆G (kcal/mol)^e^	Protein localization
**#1**	KRAS	2	c.34G > A	p.(Gly12Ser)	COSM517	Missense	0.988	Tolerated	0.644	Possibly damaging	−0.085	GTP binding site
	NRAS	3	c.181C > A	p.(Gln61Lys)	COSM580	Missense	0.991	Tolerated	0.76	Possibly damaging	−0.159	GTP binding site
**#2**	KRAS	2	c.34G > A	p.(Gly12Ser)	COSM517	Missense	0.988	Tolerated	0.644	Possibly damaging	−0.085	GTP binding site
	NRAS	2	c.38G > T	p.(Gly13Val)	COSM574	Missense	0.975	Tolerated	0.975	Damaging	4.662	GTP binding site
**#3**	KRAS	2	c.37G > T	p.(Gly13Cys)	COSM527	Missense	1	Tolerated	0.997	Damaging	2.540	GTP binding site
**#4**	KRAS	2	c.37G > T	p.(Gly13Cys)	COSM527	Missense	1	Tolerated	0.997	Damaging	2.540	GTP binding site
**#5**	KRAS	2	c.24A > G	p.(Val8Val)	COSM1360891	Silent mutation	NA	NA	NA	NA	NA	Catalytic domain
**#6**	NRAS	4	c.360G > A	p.(Leu120Leu)	Not described*	Silent mutation	NA	NA	NA	NA	NA	Catalytic domain
**#7**	KRAS	4	c.360G > A	p.(Leu120Leu)	Not described*	Silent mutation	NA	NA	NA	NA	NA	Catalytic domain
	KRAS	4	c.353G > A	p.(Cys118Tyr)	Not described*	Missense	0.988	Tolerated	0.047	Benign	0.516	GTP binding site
	KRAS	4	c.418C > T	p.(Pro140Ser)	COSM4169136	Missense	NA	NA	NA	NA	2.970	Catalytic domain
**#8**	KRAS	4	c.344G > A	p.(Gly115Glu)	Not described*	Missense	1	Tolerated	0.998	Damaging	6.967	GTP binding site
	NRAS	2	c.69A > G	p.(Leu23Leu)	*rs771113899* ^*#*^	Silent mutation	NA	NA	NA	NA	NA	
**#9**	KRAS	3	c.281G > A	p.(Arg68Arg)	Not described*	Silent mutation	NA	NA	NA	NA	NA	Catalytic domain
	KRAS	4	c.394G > A	p.(Asp132Asn)	Not described*	Missense	0.77	Tolerated	0.004	Benign	0.202	Catalytic domain
	NRAS	2	c.64C > T	p.(Gln22)*	Not described*	Stop mutation	NA	NA	NA	NA	NA	Catalytic domain
#10	NRAS	2	c.99T > G	p.(Asp33Glu)	Not described^b^	Missense	0.939	Tolerated	0.952	Damaging	0.196	Effector binding site
	KRAS	2	c.35G > T	p.(Gly12Val)	COSM520^c^	Missense	0.993	Tolerated	0.978	Damaging	−0.451	GTP binding site
#11	BRAF	15	c.1742A > G	p.(Asn581Ser)	COSM462	Missense	0.954	Tolerated	0.998	Damaging	0.595	Protein kinase domain
	NRAS	2	c.34G > T	p.(Gly12Cys)	COSM562^c^	Missense	0.935	Tolerated	0.605	Possibly damaging	−0.209	GTP binding site
#12	KRAS	2	c.40G > A	p.(Val14Ile)	COSM12722	Missense	0.999	Tolerated	0.968	Damaging	1.971	GTP binding site
	BRAF	15	c.1805C > T	p.(Ser602Phe)	Not described	Missense	0.999	Tolerated	0.916	Damaging	−1.341	Protein kinase domain
	HRAS	3	c.217C > T	p.(Arg73Cys)	Not described	Missense	1	Tolerated	0.997	Damaging	NA^f^	Catalytic domain
	MET	14	c.3050A > C	p.(Glu1017Ala)	Not described	Missense	0.889	Tolerated	0.742	Possibly damaging	NA^f^	
#13	BRAF	11	c.1396G > A	p.(Gly466Arg)	COSM253328	Missense	1	Tolerated	0.969	Damaging	3.722	ATP binding site
#14	BRAF	15	c.1781A > G	p.(Asp594Gly)	COSM467	Missense	0.998	Tolerated	0.983	Damaging	1.560	Protein kinase domain
#15	BRAF	11	c.1406G > T	p.(Gly469Val)	COSM469	Missense	1	Tolerated	0.999	Damaging	−3.553	ATP binding site
#16	BRAF	15	c.1780G > A	p.(Asp594Asn)	COSM27639	Missense	0.999	Tolerated	0.998	Damaging	−0.876	Protein kinase domain
	KRAS	2	c.38G > A	p.(Gly13Asp)	COSM532	Missense	0.988	Tolerated	0.506	Possibly damaging	3.455	GTP binding site
#17	BRAF	11	c.1406G > C	p.(Gly469Ala)	COSM460	Missense	1	Tolerated	0.83	Possibly damagin	−1.59	ATP binding site
	KRAS	2	c.35G > A	p.(Gly12Asp)	COSM521	Missense	0.99	Tolerated	0.361	Possibly damaging	−0.443	GTP binding site
#18	BRAF	11	c.1397G > A	p.(Gly466Glu)	COSM453	Missense	1	Tolerated	0.969	Damaging	3.938	ATP binding site
	KRAS	2	c.57G > T	p.(Leu19Phe)	COSM20818	Missense	0.999	Tolerated	0.999	Damaging	5.653	Catalytic domain
#19	KRAS	2	c.38G > A	p.(Gly13Asp)	COSM532	Missense	0.988	Tolerated	0.506	Possibly damaging	3.455	GTP binding site
	KRAS	4	c.436G > A	p.(Ala146Thr)	COSM19404	Missense	0.993	Tolerated	0.987	Damaging	4.757	GTP binding site
	KRAS	3	c.264A > C	p.(Lys88Asn)	Not described^b^	Tolerated	0.937	Tolerated	0.094	Benign	0.183	Catalytic domain
#20	BRAF	11	c.1406G > C	p.(Gly469Ala)	COSM460	Tolerated	1.0	Tolerated	0.835	Possibly damaging	−1.595	ATP binding site
	KRAS	4	c.351A > T	p.(Lys117Asn)	COSM28519	Tolerated	0.989	Tolerated	0.998	Damaging	0.317	GTP binding site
#21	BRAF	15	c.1801A > G	p.(Lys601Glu)	COSM478	Missense	1.0	Tolerated	0.626	Damaging	−0.276	Protein kinase domain
#22	BRAF	15	c.1799T > A	p.(Val600Glu)	COSM476	Missense	0.999	Tolerated	0.943	Damaging	0.930	Protein kinase domain
	KRAS	2	c.35G > T	p.(Gly12Val)	COSM520	Missense	0.993	Tolerated	0.978	Damaging	−0.451	GTP binding site

^a^As described in the Catalogue of Somatic Mutations in Cancer (COSMIC), available online at.

^b^Not described as somatic nor as single nucleotide polymorphism (SNP) in databases.

^c^*KRAS* and *NRAS* hotspot mutation.

^d^Mutant allele fraction (MAF).

^e^Impacts of observed mutations on protein stability, as predicted by FoldX. Negative values of ∆∆G indicate that a mutation stabilizes the protein structure relative to the WT, and positive values indicate destabilization.

^f^HRAS and MET mutations were not modelled.

## Discussion

In this study, we assessed the value of NGS-based testing to identify uncommon *KRAS*, *NRAS* and *BRAF* mutational profiles associated to *in silico* projection to evaluate their therapeutic and clinical implications in 779 samples of patients with mCRC. We identified 22 uncommon mutational profiles with a few missense variants that have not been previously reported in the literature, to our knowledge. Structural modelling of the observed missense variants in *BRAF*, *KRAS* and *NRAS* shows that most of the observed mutations can be accommodated in the protein structures without clearly adverse impacts on protein stability (Table 2). In a few cases, FoldX predicts the observed mutation to be highly destabilizing, primarily due to the introduction of interatomic clashes (e.g. *KRAS* Gly115Glu). While some such mutations may indeed lead to destabilization and inhibition of protein folding, it should be noted that the modelling procedure in FoldX does not consider backbone conformational changes. It is possible that some such mutations could be accommodated in the protein structure once backbone conformational changes are considered; an example is the *RAS* Gln61Leu mutation (not observed in this study)^14^. Despite this limitation, FoldX provides a quick, relatively accurate and parsimonious means to evaluate the impacts of mutations on protein stability.

**Figure 1:**
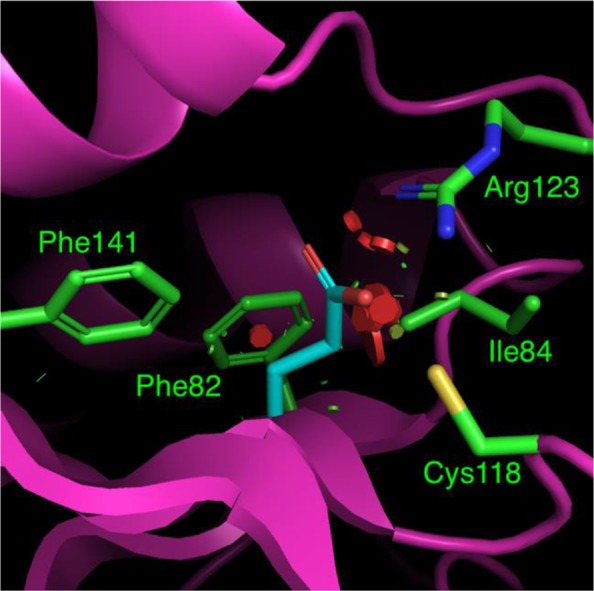
Structural model of the *KRAS* Gly115Glu variant, with key residue sidechains depicted as sticks. The Glu residue in position 115 is shown in cyan, along with neighboring residues. For clarity, only residues whose atoms contact Glu115 are shown. The mutation to Glu at this position causes severe atomic clashes, primarily with residues Ile84 and Arg123, although there is potential for hydrogen bond formation with Arg123. Clashes are indicated by colored discs drawn between atoms. The color and size of the disc reflects the severity of the clash, with wider, redder discs indicating the most severe clashes.

**Figure 2:**
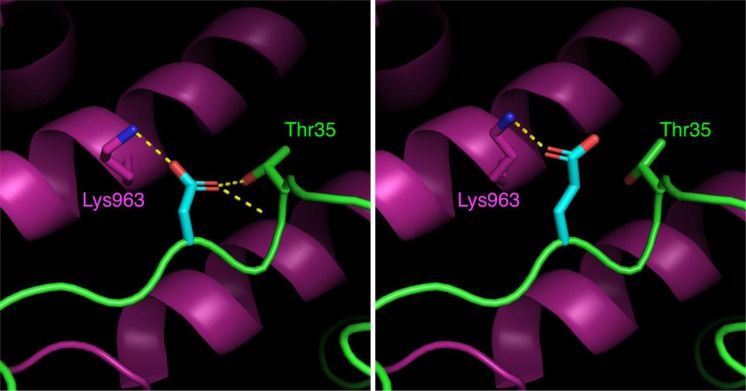
Close-up view of a section of the interface between Ras (green) and SOS (magenta) at the “catalytic” Ras-binding site of SOS^26^, with Ras residue 33 shown in cyan. Hydrogen bonds are calculated using PyMOL and shown as dashed yellow lines. The left panel shows the wild-type Asp33 residue and the right panel shows the model of Asp33Glu. The mutation causes a change in the hydrogen bonding pattern at this site. Modelling this variant at the “distal” Ras-binding site of SOS shows a similar pattern of change in the hydrogen bonding pattern. No significant interatomic clashes are created.

We now briefly describe the results of modelling the previously unreported mutations. Modelling the Gly115Glu mutation in *KRAS* (Fig. 1) suggests that this mutation has an adverse impact on protein stability. The three point mutations in *KRAS* that have not been previously reported (Lys88Asn, Cys118Tyr and Asp132Asn) are located on the protein surface and lead to minimal impacts on stability when *KRAS* is considered in isolation. The same is true of the *NRAS* Asp33Glu mutation, although this is located in the Switch 1 loop and is not directly involved in substrate binding. Examination of structures of *RAS* proteins in complex with *RASGAP* and *SOS* (PDB identifiers 1WQ1 and 1XD2, respectively) showed that most of these residues in *KRAS* and *NRAS* were not directly involved in the interaction with *RASGAP* and *SOS*. The exception is *NRAS* Asp33Glu, which may affect the interaction with *SOS* (Fig. 2)^15^. The *BRAF* Ser602Phe mutation leads to moderate stabilization relative to WT *BRAF*. This residue is located on the surface of the protein and is not in the *BRAF* dimer interface.

*BRAF* non-V600E mutations were detected in 1.43% of patients tested, which is consistent with previous published studies^16^. The predictive value of these mutations in the context of mCRC is indeterminate. However, exon 11 codon 469 *BRAF* mutations are located on the gene region coding for protein kinase function^11^. For exon 11 codon 466 mutations, the amino acid change is located within the glycine-rich loop in the kinase domain^17^. For samples #14 and #16, *BRAF* exon 15 codon 594 mutations are located on the gene region coding for protein kinase function and lead to impaired kinase activity, potentially conferring a favorable prognosis^18,19^. *BRAF* p.(Lys601Glu) (sample #21) is described as pathogenic but without published data on response to anti-EGFR mAbs therapy.

We now turn our attention to the 14 mutational profiles in our dataset with concomitant *RAS* and/or *BRAF* mutations. *KRAS* and *BRAF* mutations have been frequently described as mutually exclusive in CRC and concomitant *KRAS* and *BRAF* mutations are rare, occurring in less than 0.001% of cases^20^. In our study, in 10 samples with uncommon *BRAF* mutations, 6 are concomitant with a *RAS* mutation, and 1 *BRAF* hotspot mutation had a concomitant *RAS* mutation. The percentage of concomitant *KRAS* and *BRAF* mutations in our dataset is higher than previously described^21–27^. Since these studies only assessed *BRAF* codon 600 hotspot mutations, we may infer that hotspot *KRAS* mutations are more frequently associated with rare *BRAF* mutations than with hotspot mutations. This inference agrees with a previous study which found that patients with *BRAF* non-V600E mutations were more likely to have concomitant *RAS* mutations than patients with the *BRAF* V600E mutation^16^. Due to the infrequent observation of this phenomenon, it is actually not clear whether or not these doubly mutated tumors have a different biology and natural history than *KRAS* or *BRAF* mutant tumors. We should illustrate the potential significance of this concomitant mutation on sample #12. Sample #12 bears a *KRAS* p.(Val14Ile) mutation described as pathogenic. Whether resistance to anti-EGFR mAbs is conferred by this mutation is unknown, however codon 14 belongs to the same domain as codons 12 and 13. As with mutations on codons 12 and 13, this mutation may be associated with a clinical resistance to anti-EGFR antibodies^2^. In this case, the knowledge of this rare mutation may be significant in guiding a therapeutic decision and to explain potential resistance to anti-EGFR antibodies. However, the presence of a *BRAF* non-V600 mutation could modify response to therapy and may even lead to potential treatment resistance. Even if first observations show that *BRAF* plays only a slight role in resistance to anti-EGFR mAbs, some *BRAF* non-hotspot mutations might contribute to reduced efficacy of anti-EGFR mAbs^18,28^.

*In silico* prediction of the functional effects of the observed mutations using both sequence- and structure-based approaches suggests possible biochemical mechanisms related to uncommon mutational profile and relevant in cancer. These *in silico* results warrant further *in vivo* examination to assess the relevance of detection of non-hotspot *RAS* mutations and their implication in resistance to anti-EGFR mAbs therapy. For the *BRAF* gene, non-V600E mutations may describe a novel subtype of mCRC with better prognosis, implying potentially different treatment management strategies.

## Methods

### Samples

Data from 188 formalin-fixed paraffin embedded (FFPE) samples of histologically proven colorectal cancer tumor tissue previously published were pooled with new data from 669 FFPE samples from patients with mCRC routinely analyzed for *KRAS*, *NRAS* and *BRAF* mutations from May 2017 to May 2018 in Institut de Cancérologie de Lorraine (France)^14^. All samples were FFPE tissues from mCRC primary tumor or metastases. Determination of percentage of tumor tissue content and area for macrodissection were based on examination of hematoxylin-eosin stained sections by a senior pathologist. All patients involved in this study gave their informed consent for the research of *KRAS*, *NRAS* and *BRAF* mutation. The experimental protocols of these study have been approved by the ethical and scientific board of Institut de Cancérologie de Lorraine. All methods were performed in accordance with the relevant guidelines and regulations. All data were anonymized prior to analysis.

### DNA extraction and quality assessment

For all samples, DNA was extracted as previously described using QIAamp DNA FFPE Tissue Kit (Qiagen, Hilden, Germany)^14^. After extraction of the 669 new samples, TruSeq FFPE DNA Library Prep QC kit (Illumina, San Diego, USA) and qPCR using Cobas z480 (Roche Diagnostics, Meylan, France) were used for quality DNA assessment. Cycle quantification (Cq) values were calculated using LightCycler® 480 Software W UDF 2.0.0 (Roche Diagnostics). For samples showing a ΔQC score lower than 6, DNA libraries were then prepared using TruSeq® Custom Amplicon Library Preparation Kit v1.5 (Illumina). Fifty-three samples failed to yield sufficient DNA quality and 616 samples qualified for DNA library preparation.

### DNA library preparation and sequencing

For the first 188 samples, library preparation and DNA sequencing were performed using the GS Junior deep pyrosequencing system as previously described^14^. Library preparation was not possible for 11 samples.

For the 616 new samples, libraries were prepared using the TruSeq® Custom Amplicon Library Preparation Kit v1.5. This kit consists of two separate oligo pools (CATA and CATB) and allow the full exon analysis of 16 genes: *AKT1 (*exon3)*, ALK (*exons 23 to 25)*, BRAF* (exons 11 and 15)*, EGFR* (exons 18 to 21)*, ERBB2 (*exon 20)*, ERBB4* (exons 10 and 12)*, FGFR2* (exons 7, 12 and 14)*, FGFR3* (exons 7, 9 and 14)*, HRAS* (exons 2, 3 and 4)*, KIT* (exons 8, 9, 11, 13, 17 and 18)*, KRAS* (exons 2, 3 and 4)*, MAP2K1* (exon2)*, MET* (exons 2 and 14 to 20)*, NRAS* (exons 2, 3 and 4)*, PDGFRA* (exons 12, 14 and 18) *and PIK3CA* (exons 10 and 21). The two oligo pools were hybridized to DNA samples. The specific hybridized targets were ligated, extended and PCR amplified with adaptors containing index with specific barcode sequences. Two complementary libraries were generated by targeting the forward and reverse DNA strands. The PCR-amplified amplicon libraries obtained were then purified using AMPure XP beads in order to remove non-specific products and reaction components.

Library DNA concentrations were quantified using Qubit 3.0 Fluorometer (ThermoFisher Scientific Inc, Massachusetts, USA) and their quality was assessed on Fragment Analyzer (Advanced Analytical, Ankeny, USA) using the Standard Sensitivity NGS Fragment Analysis Kit (Advanced Analytical). PCR product sizes have to be around 260 base pairs in length. All 616 validated libraries were normalized to enable similar amplification and sequencing levels for each sample library in the same run. Sequencing was performed according to the manufacturer’s instructions. All libraries were pooled before sequencing on the MiSeq instrument (Illumina). Sequencing data analysis was performed on Sophia DDM® software (Sophia genetics, Saint Sulpice, Switzerland). Reference sequences NM_033360.2 for *KRAS*, NM_002525.4 for *NRAS* and NM_004333.5 for *BRAF* were used for alignment and variant calling. Fourteen samples had insufficient coverage to be interpretable and 602 samples had interpretable sequencing data.

### Uncommon mutational profiles

Uncommon mutational profiles were defined as i) a concomitant *KRAS* and *NRAS* hotspot mutations, or ii) a *KRAS*, *NRAF* or *BRAF* non-hotspot mutation (associated or not associated with other mutations). A threshold of 1% allele frequency has been reported clinically relevant for *KRAS* mutations linked with lack of response to anti-EGFR therapy^29^.

For samples with an uncommon mutational profile, mutational status of *MAP2K1* and *HRAS* genes (available in our gene panel and implicated in the MAPkinase pathway) have been also identified.

### *In silico* prediction of mutation impact

We used PolyPhen-2 (Polymorphism Phenotyping) and SIFT (Sorting Intolerant from Tolerant) scores to predict mutation impact on the protein, as well as FoldX to model the observed variants in protein structures^30–34^. PolyPhen-2 score predicts the possible impact of an amino acid substitution on the structure and function of a human protein. PolyPhen combines amino acid composition analysis in multiple sequence alignments with information from solved protein structures (where available). Sequence composition is evaluated using the Position Specific Independent Counts (PSIC) tool, which calculates sequence profiles^35^. Differences in the profiles calculated by PSIC for the different variants at a site are indicative of damaging impact. In cases where structural data are available, the impact of a mutation is assessed by considering physicochemical properties such as residue size and hydrophobicity, and the maintenance of contacts with ligands, metals or other interacting proteins. Sequence- and structure-based features are combined to produce predictions using a naïve Bayes classifier. PolyPhen-2 produces scores between 0 and 1 along with annotations of whether the mutation is predicted to be benign or damaging.

SIFT also predicts whether an amino acid substitution affects protein function. SIFT scores are used to predict the damaging effect of nucleotide substitutions and frame shifts (insertions/deletions) on protein function based on the maintenance of amino acid composition in alignments of the target sequence with closely related sequences. The SIFT server assigns scores for each residue from 0 to 1, where mutations with a score of ≤0.05 are predicted to not be tolerated, and mutations with score >0.05 are predicted to be tolerated^36^.

SIFT and PolyPhen-2 scores were determined using Sophia DDM® software (version 5.0.7). Structural modelling of observed missense mutations was carried out using FoldX version 4^33,34^. FoldX estimates the impact of point mutations on the folding energy or stability of the protein using rigid-backbone modelling and a classical forcefield whose parameters are trained to reproduce experimental observations of mutational impacts on folding energy. Starting from the wildtype structures for *KRAS*, *BRAF* and *NRAS* (PDB identifiers 4OBE, 5VAM and 5UHV, respectively), the missense mutations observed in these proteins were modeled using the FoldX BuildModel tool, and the structural and energetic impacts of each mutation were assessed as the predicted change in folding energy (∆∆G). Synonymous (silent) and stop mutations were not modeled. All FoldX modelling results are available at 10.5281/zenodo.1467311.

### Protein domain impacted

*KRAS* and *NRAS* protein domains impacted by the different mutations described were identified using UCSF Chimera^37^. The COSMIC database was used to identify protein domains impacted by *BRAF* mutations^38^.

## Data Availability

The authors confirm that the data supporting the findings of this study are available within the article. The FoldX modelling data that support the findings of this study are available at 10.5281/zenodo.1467311.
